# A Case Report of a Pregnant Woman With Type 2 Diabetes Mellitus Using Dulaglutide During the First Trimester of Pregnancy

**DOI:** 10.7759/cureus.44644

**Published:** 2023-09-04

**Authors:** Adel Alghamdi, Abeer Alsaeddi, Hashem Malki, Ameerah Alsaedi

**Affiliations:** 1 Diabetes and Endocrinology, Diabetes Center of Hera General Hospital, Makkah, SAU; 2 Family Medicine, Al-Awali Primary Health Care, Makkah, SAU; 3 Endocrinology, Diabetes Center of Al-Noor Hospital, Makkah, SAU

**Keywords:** diabetes in pregnancy, pregnant females, glp-1 agonist, fetal outcomes, diabetes type 2

## Abstract

Among women of childbearing age, type 2 diabetes mellitus (T2DM) is becoming more prevalent, increasing the likelihood of abortion, congenital anomalies, and neonatal death. Dulaglutide has not been adequately studied to determine if it causes birth defects or miscarriages during pregnancy. According to animal studies, the fetus is at risk from the use of dulaglutide during pregnancy. We report the case of a 39-year-old woman with T2DM who used dulaglutide (1.5 mg/week) along with glargine and aspart before conception. During the third month of pregnancy, she was seen in the clinic for the first time during which dulaglutide was stopped and basal-bolus insulin therapy was retained with dosing titration. The newborn was a male with a normal birth weight for his gestational age. Dulaglutide did not affect development. No minor or major malformations were noted in the fetus except for mild bilateral renal pyelectasis. Moreover, no maternal or fetal complications were observed. It is not possible to ascertain the safety of glucagon-like peptide-1 receptor agonists in pregnancy, despite the normal outcome in the present pregnancy; however, the data described here may be of value in further considering this issue.

## Introduction

Among women of childbearing age, type 2 diabetes mellitus (T2DM) is becoming more prevalent, increasing the likelihood of abortion, congenital anomalies, and neonatal death [1]. The incidence of obesity among US adults continues to increase and is strongly associated with the risk of diabetes [2]. The effects of glucose-lowering medications on weight should be considered when choosing them for T2DM patients who are overweight or obese [3,4]. This is an important issue to be considered when treating pregnant T2DM patients. Dulaglutide is a glucagon-like peptide-1 receptor agonist (GLP-1 RA) approved by the Food and Drug Administration (FDA) for the treatment of T2DM in adults. A higher efficacy approach is now recommended to achieve glycemic control and weight management [4]. Dulaglutide is an option with high efficacy [4].

Dulaglutide has not been adequately studied to determine if it causes birth defects or miscarriages during pregnancy [5]. According to animal studies, the fetus is at risk from the use of dulaglutide during pregnancy; hence, dulaglutide use in pregnancy has not been approved by the FDA [5].

Dulaglutide administration during organogenesis resulted in early embryonic death, fetal growth reduction, and fetal abnormalities in pregnant rats [5]. Major fetal abnormalities were observed in pregnant rabbits administered dulaglutide during organogenesis [5]. Due to the pharmacology of dulaglutide, adverse effects on embryos and fetuses were observed in animals associated with decreased maternal weight and food consumption [5].

Due to unplanned pregnancies in women with T2DM, eight cases have been reported in the literature [6]. In one study, during the first trimester of pregnancy, a fetus was exposed to dulaglutide [6]. Two women chose to terminate their pregnancies electively, while live births were achieved in the other six pregnancies without neonatal complications [6]. Two pregnant women developed hypertension, with one developing cholestasis and hyperglycemia during pregnancy [6].

## Case presentation

We report the case of a 39-year-old woman with T2DM for 19 years. She was on dulaglutide (1.5 mg/week) plus glargine 20 IU daily and aspart 10 IU three times before conception. The patient was overweight (body mass index = 29.6 kg/m^2^) without micro and macrovascular complications. She was seen for the first time in the diabetic clinic at the gestational age (GA) of 13 weeks. Blood glucose was uncontrolled as the patient was non-compliant with treatment (HbA1c = 9%). Therefore, dulaglutide was stopped and she was retained only on basal-bolus insulin therapy with dosing titration as dulaglutide is not approved during pregnancy. Moreover, she received diet counseling to manage diabetes during pregnancy. At 22 weeks of GA, because blood glucose readings remained high, the insulin dose was increased to glargine 36 IU and aspart to 22 IU three times daily.

**Figure 1:**
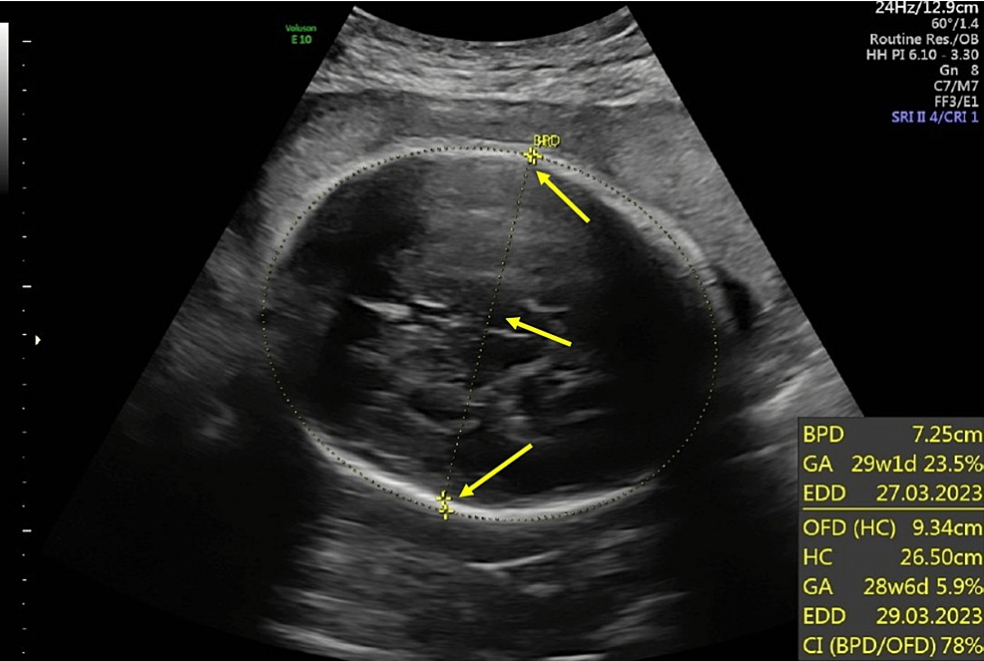
Biparietal diameter. BPD: biparietal diameter; GA: gestational age; EDD: expected date of delivery; OFD: occipitofrontal diameter; HC: head circumference; CI: cephalic index

**Figure 2:**
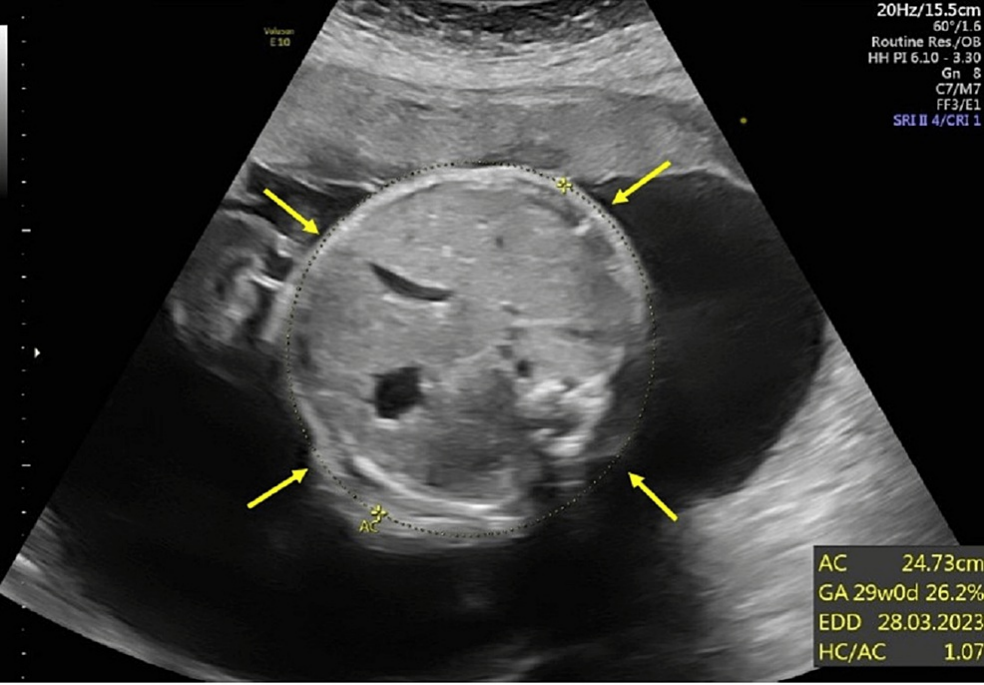
Abdominal circumference. AC: abdominal circumference; GA: gestational age; EDD: expected date of delivery; HC: head circumference

**Figure 3:**
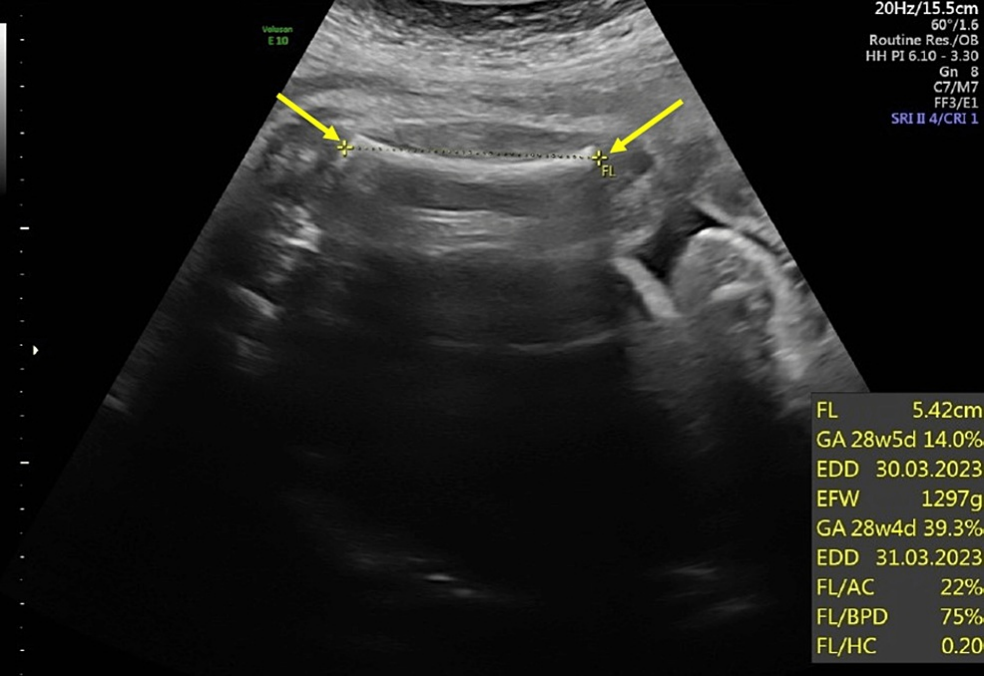
Femur length. FL: femur length; GA: gestational age; EDD: expected date of delivery; EFW: estimated fetal weight; AC: abdominal circumference; BPD: biparietal diameter; HC: head circumference

**Figure 4:**
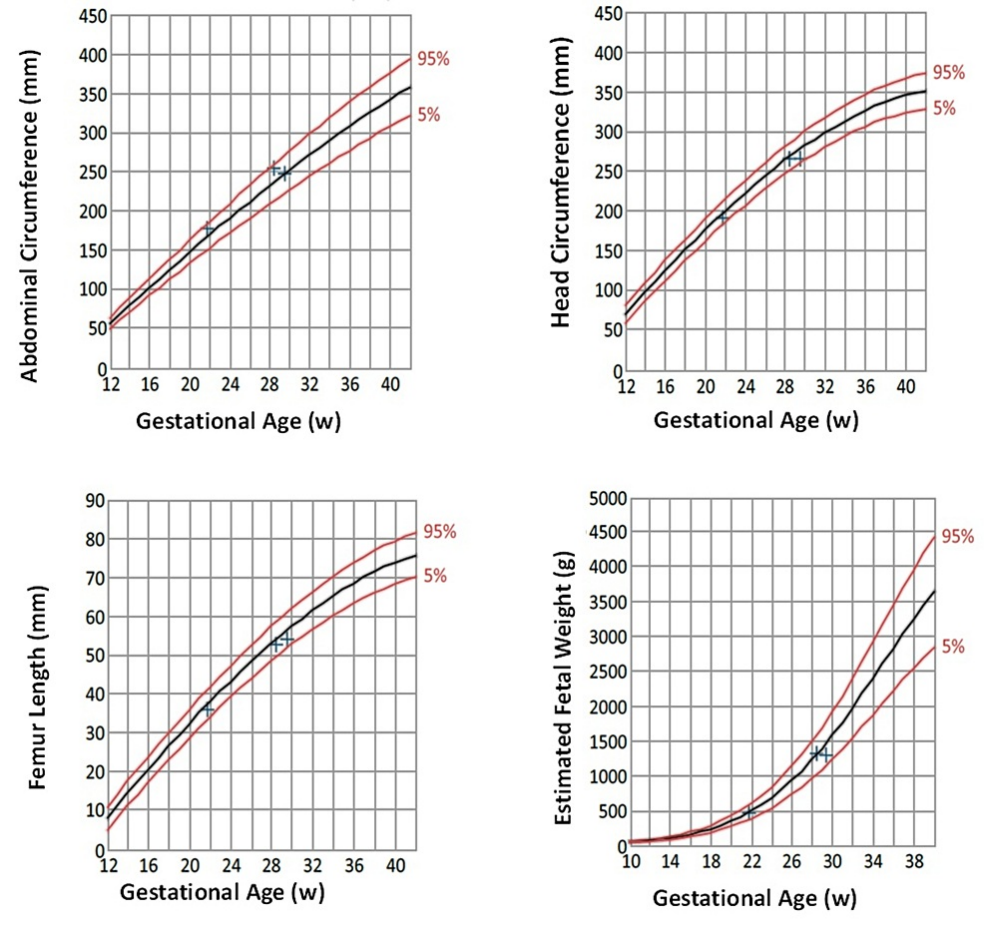
Fetal growth chart of abdominal circumference, head circumference, femur length, and estimated fetal weight.

**Figure 5:**
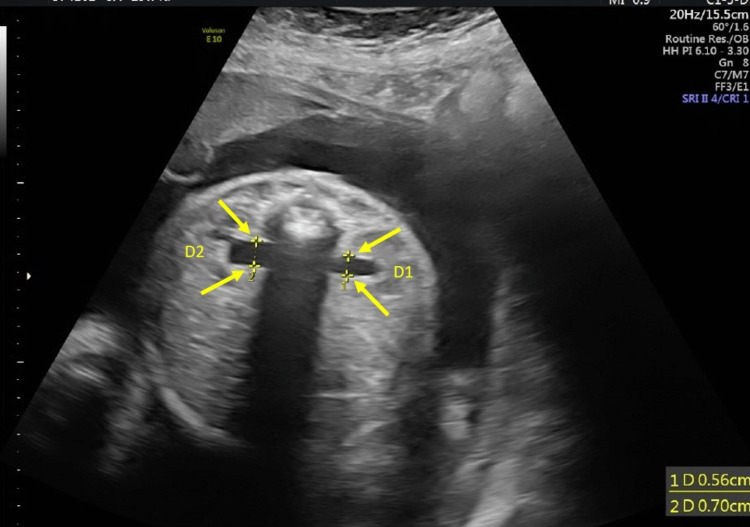
Mild bilateral renal pyelectasis (6 and 7 mm).

During pregnancy, routine fetal biometry ultrasounds were done measuring biparietal diameter (BPD), head circumference (HC), abdominal circumference (AC), and femur length (FL). All fetal biometry measurements were normal and did not show any fetal growth abnormalities. The amniotic fluid index was also assessed and was within normal limits. A fetal anatomy survey was done on 28 weeks of GA. The fetal biometry during the survey showed that BPD was 72.5 mm (Figure 1), AC was 247.3 mm (Figure 2), and FL was 543 mm (Figure 3). The parameters of the fetal growth chart were within the normal limits (Figure 4). No gross anomaly was seen on the fetal anomaly survey report but there was mild bilateral renal pyelectasis (6 and 7 mm) (Figure 5). Due to mild bilateral renal pyelectasis, non-invasive prenatal testing (NIPT) was done to look for chromosomal disorders and the result was negative. The patient underwent an elective cesarean section at 38 weeks four days, as the patient had a history of three previous cesarean deliveries. The newborn was a male with a weight of 3,100 g (23rd percentile), a length of 50 cm (48th percentile), and an HC of 33 cm (10th percentile). The baby’s weight was normal for his GA. No neonatal morbidities or congenital anomalies were seen except for the presence of mild bilateral renal pyelectasis. The mother did not develop any complications during cesarean delivery.

## Discussion

In our case, dulaglutide did not affect fetal growth. No malformations were seen, and there were no complications, neither in the mother nor the fetus, except for the presence of mild bilateral renal pyelectasis. Usually, fetal renal size is smaller in mothers with T2DM compared to non-diabetic mothers [7]. The presence of renal pyelectasis and advanced maternal age (>35 years old) are risks for developing Down syndrome [8]. In our case, NIPT was done, and the result was negative. No data is available regarding the effects of dulaglutide on fetal development or renal fetal size. We cannot consider the safety of using GLP1-RA during pregnancy. However, these data can contribute to improving our knowledge about the effects of GLP1-RA on fetal growth.

Unplanned pregnancy, as in our case, is associated with suboptimal diabetic control, which is a significant factor contributing to poor pregnancy outcomes in these women [9]. Moreover, these women can be managed with effective drugs for controlling diabetes and weight, but they may have side effects on pregnancy [5,10]. Diabetic women should learn about the importance of controlling blood glucose before getting pregnant [11,12]. They should also learn about the contraindicated diabetic drugs during pregnancy. Furthermore, all diabetic women should be educated if they plan a pregnancy to only use safe and well-studied diabetic medications for pregnancy and the fetus before getting pregnant [11,12].

## Conclusions

Despite the normal outcome in our case without any complications apart from renal pyelectasis, the safety of GLP1-RA is not established, and more research is required to confirm or establish its safety. More data are needed to consider its safety.

## References

[1] Coton SJ, Nazareth I, Petersen I (2016). A cohort study of trends in the prevalence of pregestational diabetes in pregnancy recorded in UK general practice between 1995 and 2012. BMJ Open.

[2] Mokdad AH, Ford ES, Bowman BA, Dietz WH, Vinicor F, Bales VS, Marks JS (2003). Prevalence of obesity, diabetes, and obesity-related health risk factors, 2001. JAMA.

[3] ElSayed NA, Aleppo G, Aroda VR (2023). 8. Obesity and weight management for the prevention and treatment of type 2 diabetes: standards of care in diabetes-2023. Diabetes Care.

[4] ElSayed NA, Aleppo G, Aroda VR (2023). 9. Pharmacologic approaches to glycemic treatment: standards of care in diabetes-2023. Diabetes Care.

[5] Eli Lilly and Company (2020). Eli Lilly and Company. Trulicity US [2020] prescribing information. https://pi.lilly.com/us/trulicity-uspi.pdf.

[6] Burlina S, Dalfrà MG, Caprino R, Lapolla A (2023). A case report on use of dulaglutide during the first weeks of pregnancy in woman affected by type 2 diabetes mellitus. Acta Diabetol.

[7] Pylypjuk CL, Day C, ElSalakawy Y, Reid GJ (2022). The significance of exposure to pregestational type 2 diabetes in utero on fetal renal size and subcutaneous fat thickness. Int J Nephrol.

[8] Corteville JE, Dicke JM, Crane JP (1992). Fetal pyelectasis and Down syndrome: is genetic amniocentesis warranted?. Obstet Gynecol.

[9] Lapolla A, Dalfrà MG, Fedele D (2008). Pregnancy complicated by type 2 diabetes: an emerging problem. Diabetes Res Clin Pract.

[10] Brown E, Heerspink HJ, Cuthbertson DJ, Wilding JP (2021). SGLT2 inhibitors and GLP-1 receptor agonists: established and emerging indications. Lancet.

[11] Murphy HR, Howgate C, O'Keefe J (2021). Characteristics and outcomes of pregnant women with type 1 or type 2 diabetes: a 5-year national population-based cohort study. Lancet Diabetes Endocrinol.

[12] Lapolla A, Dalfrà MG, Di Cianni G, Bonomo M, Parretti E, Mello G (2008). A multicenter Italian study on pregnancy outcome in women with diabetes. Nutr Metab Cardiovasc Dis.

